# Refractory perioral dermatitis treated with baricitinib: A case report and literature review

**DOI:** 10.1016/j.jdcr.2026.06.004

**Published:** 2026-06-12

**Authors:** Kyung-Gu Chung, Min Kyung Shin

**Affiliations:** Department of Dermatology, Kyung Hee University College of Medicine, Kyung Hee University Hospital, Seoul, Korea

**Keywords:** baricitinib, perioral dermatitis

## Introduction

Perioral dermatitis (POD) is a chronic, relapsing inflammatory skin condition characterized by erythematous papules and pustules typically localized around the perioral and periocular areas. POD has been most frequently reported in young women and children, although its exact annual incidence remains unclear.^1^

The pathogenesis of POD is not fully understood, but several mechanisms have been proposed, including skin barrier dysfunction, dysregulation of innate immunity, and microbial dysbiosis.^2^

Among known triggers, long-term topical corticosteroid use is one of the most significant, often provoking paradoxical rebound flares and perpetuating disease chronicity.

Several therapeutic options have been suggested based on expert consensus. Topical corticosteroids should first be discontinued, and alternative topical agents such as antibiotics or calcineurin inhibitors are widely used. In more severe cases, oral antibiotics including macrolides and tetracyclines are frequently prescribed. However, despite these therapeutic options, many patients respond poorly or experience treatment-related adverse events, making management challenging.

Recently, several case reports have described clinical improvement with Janus kinase (JAK) inhibitors in refractory POD unresponsive to conventional treatments.^3^^,^^4^ In this context, we report a case of refractory POD successfully treated with baricitinib.

## Case report

A 23-year-old female patient presented with a 2-month history of an itchy facial rash primarily involving the periocular and perioral areas. The eruption developed without any prior use of topical corticosteroids. After the onset of the eruption, the patient reported that she had been treated with topical corticosteroids (prednicarbate) and a topical calcineurin inhibitor (tacrolimus), without clinical improvement. The patient had no underlying conditions such as asthma or atopic dermatitis.

**Fig 1:**
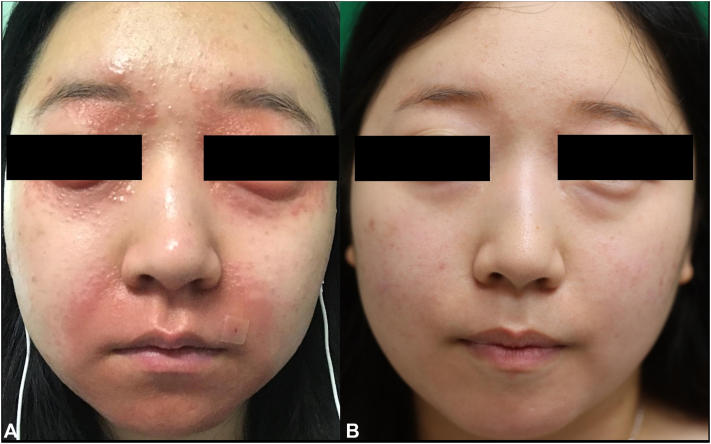
The skin lesions before and after baricitinib therapy. **A,** Initial presentation; erythematous patches and grouped follicular papules were observed around the eyes, nose and mouth **(B)** Two months after baricitinib treatment; complete resolution was observed.

On physical examination, diffuse erythematous patches were observed around the eyes, nose, and mouth, with grouped erythematous follicular papules overlying the affected areas (Fig 1, *A*). Based on the characteristic distribution of lesions in the periorificial areas, the absence of comedones, and the clinical history, a clinical diagnosis of POD was made. In view of the significant disease burden and possible coexisting eczematous component, cyclosporine was initiated as an adjunctive off-label therapy. Initial treatment consisted of oral minocycline (50 mg/day), cyclosporine (100 mg/day), and antihistamines for 2 months; although partial improvement was observed, the condition relapsed frequently. Subsequently, minocycline was switched to low-dose isotretinoin (10 mg every other day), while cyclosporine and antihistamines were maintained. This combination therapy was continued for an additional 2 months without significant improvement. During the 4-month treatment period, the cyclosporine dose was adjusted between 50 and 150 mg/day depending on disease activity. Given the inadequate response to multiple conventional treatment approaches, treatment was switched to baricitinib. Baricitinib treatment commenced at a dosage of 4 mg per day. After 1 month of therapy, the patient showed remarkable improvement in pruritus and facial rash. The dose was then reduced to 2 mg daily and continued for an additional month, after which the lesions fully resolved and baricitinib was discontinued (Fig 1, *B*). The patient has not reported any relapse of her condition during the 4-month follow-up period after discontinuation of baricitinib. No treatment-related adverse events were reported during the whole treatment period.

## Discussion

### POD

• POD is a multifactorial inflammatory disorder that often becomes chronic and recurrent, leading to therapeutic challenges in some patients.
• Although dysregulated innate immunity and impaired barrier function have been suggested as contributors to these refractory cases, the exact pathogenesis of POD remains incompletely understood.

### Current treatment landscape

• Currently, there are no therapies approved by the U.S. Food and Drug Administration specifically for POD.
• Management relies on withdrawal of triggering factors and off-label use of topical and systemic agents; however, some patients experience persistent disease with poor response to conventional therapies.
• Emerging evidence suggests a potential role for JAK inhibitors in the management of refractory POD.^3^^,^^4^

### JAK inhibitors and POD

• Interestingly, recent literature describes 2 contrasting observations regarding JAK inhibitors in POD.
• Several reports have demonstrated significant improvement in refractory POD with JAK inhibitors, likely through inhibition of JAK1/2-mediated signaling pathways involving interferon-γ and other proinflammatory cytokines that may contribute to its pathogenesis (Table I).^3^^,^^4^

• Conversely, other studies have paradoxically documented POD or rosacea-like eruptions developing after JAK inhibitor therapy (Table II).^5^, ^6^, ^7^

**Table I:** Published cases of perioral dermatitis successfully treated with Janus kinase inhibitors

Source	Age/sex	Underlying disease	JAK inhibitor	Indication for JAK inhibitor	JAK inhibitor effect on POD	Outcome
Teng Y et al,^3^ 2023	26/F	None	Abrocitinib	Treatment for refractory POD	Skin lesions and pruritus had almost completely disappeared	Sustained POD remission
Tran SS et al,^4^ 2024	18/F	Atopic dermatitis (AD), asthma	Ruxolitinib (topical)	Treatment for refractory POD	POD had completely resolved, with normalization of skin pigmentation	Sustained POD remission
Present case, 2025	23/F	None	Baricitinib	Treatment for refractory POD	POD had completely resolved.	Sustained POD remission

*JAK*, Janus kinase; *POD*, perioral dermatitis.

• One proposed hypothesis for this paradoxical phenomenon is that JAK inhibitors may suppress interferon-mediated antimicrobial immunity, resulting in alterations of the skin microbiome or Demodex overgrowth.^5^^,^^6^^,^^8^
• These conflicting observations suggest that the immunologic pathways involved in POD may be complex and may differ among individual patients.

**Table II:** Published cases of perioral dermatitis or rosacea-like eruptions developing after Janus kinase inhibitor therapy

Source	Age/sex	Underlying disease	JAK inhibitor	Indication for JAK inhibitor	JAK inhibitor effect on POD	Outcome
Paolino G et al,^5^ 2024	26/F	Atopic dermatitis (AD)	Upadacitinib	Treatment for severe AD	Papulopustular POD developed, accompanied by a burning sensation	Upadacitinib was discontinued and the patient was treated with doxycycline and topical clindamycin
Paganini C et al,^6^ 2025 (Case 1)	52/M	AD	Upadacitinib	Treatment for severe AD	Papulopustular lesions on the face (cheeks and forehead) developed	Upadacitinib was discontinued and the patient was treated with doxycycline, oral metronidazole and topical metronidazole
Paganini C et al,^6^ 2025 (Case 2)	42/M	AD	Upadacitinib	Treatment for severe AD	Rosacea-like eruption with erythematous papulopustules developed	Upadacitinib was discontinued and the patient was treated with doxycycline
Neumann K et al,^7^ 2021	32/M	Ulcerative colitis (UC)	Tofacitinib	Treatment for UC	Severe papulopustular dermatitis developed	Tofacitinib was discontinued and the patient was treated with doxycycline, oral corticosteroids, and topical metronidazole

*JAK*, Janus kinase; *POD*, perioral dermatitis.

### Clinical implications and future directions

• In our case, baricitinib led to sustained remission, supporting a potential therapeutic role for targeted cytokine modulation in refractory POD.
• Because JAK inhibitor-induced POD has also been reported, careful patient selection and close monitoring remain essential.
• Given the heterogeneous pathogenesis of POD, it is plausible that individual immunologic endotypes may determine whether JAK inhibition ameliorates or, conversely, precipitates disease activity.
• Further studies are needed to elucidate the precise mechanisms by which JAK inhibitors influence POD and to identify biomarkers that can help define the subset of patients most likely to benefit from JAK inhibitors.

## Conflicts of interest

None disclosed.
